# Ureteroscopic Laser Treatment of Upper Urinary Tract Urothelial Cell Carcinomas: Can a Tumour Free Status Be Achieved?

**DOI:** 10.1155/2013/429585

**Published:** 2013-09-12

**Authors:** Christos Kalaitzis, Athanasios Zisimopoulos, Stilianos Giannakopoulos, Stavros Touloupidis

**Affiliations:** ^1^Department of Urology, University of Thrace, 68100 Alexandroupolis, Greece; ^2^Department of Nuclear Medicine, University of Thrace, 68100 Alexandroupolis, Greece

## Abstract

*Introduction*. In cases of anatomic or functional single kidney with urothelial tumours of the upper urinary tract, the endoscopic laser ablation has proven efficacious. Based on the knowledge that low-grade, low-stage upper tract transitional cell carcinomas rarely progress to invasive lesions, indications for endoscopic laser ablation have expanded to include patients with bilateral functioning kidneys and low-grade tumours. The question that remains to be answered is whether endoscopic laser ablation has the ability to completely eradicate upper urinary tract tumours. *Methods*. We performed in 25 patients in a period of 11 years 288 ureteroscopies and, if needed, laser ablation of upper urinary tract tumours in imperative indication. *Results*. In 32% of the patients the cancer remained even after several laser sessions. 64% of patients were tumour free after one or more laser sessions but remained clear only for the next 3 months. Only 1 patient was tumour free for a period of 68 months after 1 session of laser treatment. The procedure had low complication rates. *Conclusion*. The laser technology and the introduction of small diameter semirigid and flexible ureteroscopes made ablation of upper urinary tract tumours possible and safe. Nevertheless a complete resection of the carcinomas is rarely possible.

## 1. Introduction

Upper urinary tract urothelial carcinomas (UTUCs) occur with an incidence of 1-2/100.000 in Western countries and comprise up to 5–10% of all urothelial carcinomas [1]. Radical nephroureterectomy (RNU) with bladder cuff excision is considered the gold standard treatment. However this procedure requires an intact contralateral kidney. In cases of a solitary kidney, bilateral kidney involvement by tumour or in cases of chronic renal insufficiency, organ preservation is crucial, and kidney sparing approaches are indicated (imperative indication). 

The demonstrated lack of aggressiveness of low-grade UTUCs together with the advent of improved ureterorenoscopes and advanced lasers technology has pushed the elective indications of endoscopic ablation of UTUC to include patients with upper urinary tract urothelial tumours and normal contralateral kidney function [1, 2] (nonimperative or elective indication). The European Urology Guidelines recommend the endoscopic treatment only for patients with low-grade and low-stage urothelial tumours [1]. The success rate of ureteroscopic therapy for grade 1 and grade 2 UTUCs is similar to that of bladder lesions managed by transurethral resection [3].

Endoscopic treatment of UTUCs can be performed ureteroscopically or percutaneously.

The percutaneous approach is reserved for large tumours and is associated with greater morbidity with transfusion rates of 17% [4].

In the literature, 5-year survival rates of 100% for G1 tumours [5] versus 32–38% for G3 [6–8] have been reported. 5-year disease specific survival (DSS) has been reported to range between 81 and 100% for low-grade transitional cell carcinomas (TCCs) versus 69–86% for high grade disease [9–11] despite the inability of 32% to resect completely UTUCs after multiple ureteroscopic procedures [12]. The recurrence rate of UTUCs after endoscopic procedures was found to range from 25.7% to 44.4% [13]. Tumour grade depended recurrence rate was 40% for low-grade tumours versus 76%–88% for high grade tumours [12, 14].

In our prospective study we managed 25 patients with UTUCs ureteroscopically in imperative indication, and we examine the ability to resect tumours completely after one or more sessions.

## 2. Methods

25 patients, 13 men (52%) and 12 women (48%), underwent endoscopic treatment for UTUCs ureteroscopically in imperative indication. The average age of the patients was 71 ± 9 years. 10 patients had a single kidney (4 of them not due to tumour and 6 after nephroureterectomy of the contralateral kidney for UTUCs). 6 patients had comorbidity, 3 bilateral disease, and 6 borderline kidney insufficiency. In all patients we performed ureterorenoscopy under general anaesthesia every 3 months. Semirigid and flexible ureteroscopes are used. Before each ureterorenoscopy, lavage cytology specimens were taken, and a contrast medium was given retrogradely.

Before laser treatment, tumour biopsies or biopsies from the suspect area were conducted. Only when inspection, retrograde imaging, lavage cytology, and biopsy were normal, and the renal unit was considered tumour free.

Holmium laser device was used as the energy source (Ho:YAG laser). For energy delivering we used a 365 *μ*m fibre. We worked with energy of 1.0−1.5 J and pulse between 6 and 8 Hz. The surgery time amounted from 30 up to 150 minutes (average 74.8 minutes) while the length of intermediate inpatient stay was 4 days. After each ureterorenoscopy a DJ stent was inserted for 2 weeks. 

## 3. Results

14 (56%) patients had G1 urothelial tumour, 5 (20%) patients had G1-2, 4 (16%) patients G2, and 2 patients (8%) a G3 urothelial tumour. In 14 (56%) patients tumour was solitary and in 11 (44%) multifocal. Six (24%) patients had never urinary bladder tumour before, 10 (40%) patients had urinary bladder tumour in the history, 1 patient had synchronously kidney pelvis and bladder tumour, and 7 (28%) patients developed bladder tumour during the followup.

With a time range of 3 to 68 months (median 6 months), 8 (32%) patients did not manage to get tumour free even after several laser sessions. Seven of them (68%) had multifocal tumour and 1 patient a G3 tumour > 2.5 cm. 

16 (64%) patients were tumour free after one or several laser treatment sessions only for the next 3 months. At the next ureteroscopy 3 months later, these patients showed a recurrence and had to be treated. After 68 months and 1 session laser treatment, only 1 patient was tumour free. Patient had a solitary G1 tumour < 1.5 cm. 

Thus, 24 of 25 patients (96%) relapsed during followup of 68 months. In one patient we performed, after 2 years, a nephroureterectomy because of local disease progression (CT imaging). This patient had a G3 urothelial tumour of the renal pelvis.

During followup of 68 months, we performed a total of 288 ureteroscopies in 25 patients in a period of 11 years (2000–2011). In 11 of 288 ureteroscopies (3.8%), there was a bleeding required blood transfusion. In a patient, it came to a septicaemia with *Klebsiella*. This patient had to be treated in intensive care. In a patient with renal-pelvic tumour, it came to ureteral demolition. Ureter was replaced by intestinal segment. The followup was still possible through the bowel segment. Three patients (12%) developed ureteral strictures that were not due to tumour. In these patients, we permanently left the DJ stent, and we changed it at each session (Table 1). 

During followup of 68 months (median 6 months), 1 patient with a G3 tumour died of pulmonary metastases and 1 diabetic patient of the consequences of ischemic brain insult.

## 4. Discussion

The majority of the trials reported no progression in grade and stage of low-grade upper tract urothelial tumours during followup of average 32 months [2]. Disease specific mortality for low-grade tumours was zero [2]. In the study of Martinez-Pineiro no deep tumour invasion in all patients with low-grade lesions treated ureteroscopically was also found [15]. The situation is different in patients with high grade UTUCs. While the 5-year survival rate for low grade tumours is 100%, it is only 32% up to 38% for high grade UTUCs [9, 10, 15, 16].

Biopsies of UTUCs are not always able to determine the right grade of the tumours. A study conducted by the Mayo Clinic, in which the grade of the biopsies from UTUCs was compared with the grade of the same tumours in kidney preparation after ureteronephrectomy, showed that 96% of G1 and 40% of G2 UTUCs in the biopsy were upgraded in the nephrectomy preparation. In 25% of the cases biopsy was unable to establish diagnosis [17]. Even if a G2 UTUC be upgraded is simple a G3 UTUC. The recurrence rate of high grade tumours is 76% up to 88% compared with 40% for low-grade tumours [12, 14].

Keeley found that the recurrence rate is also dependent on the size of the primary tumour, and tumours >1.5 cm had a significantly higher rate of local recurrence [13]. In summary, disease recurrence is significantly influenced by initial tumour characteristics: localisation, size, grade, multifocality, and the concomitant presence or absence of bladder tumour. Unfavorable are tumour size > 2 cm, location in kidney pelvis, multifocality, and at least 3 previous resections of bladder tumours [13, 18, 19]. Although another study has shown that localisation of the tumour is less important for recurrence, in this study local recurrence rate was 33% for renal-pelvic tumours and 31% for ureteral tumours [20].

In spite of an increased recurrence rate, ureteroscopic treatment of UUTT does not impair overall survival [21]. However, it must be said that the average age of patients in all studies was about 70 years. In our study, we performed a total of 288 ureteroscopies in 25 patients with UTUCs in a period of 11 years. 32% of the patients were never tumour free after multiple ureteroscopic laser procedures. This is exactly the result of other studies [13, 15, 19]. After one or more laser treatment sessions, 16 (64%) patients were at the next control after 3 months of being tumour free. However in the course, they showed a tumour recurrence. In only one patient we reached a tumour free status for 68 months after 1 laser session. This patient had a solitary G1 ureteral tumour < 1.5 cm. Complication rate of ureteroscopic management of UTUCs is low (Table 1). Reported stricture rate in large series has ranged from 5% to 14% [22]. Ureteral strictures are not always a result of the medical act. Daneshmand et al. found that 40% of patients with strictures after ureteroscopic treatment had recurrent UTUCs [23]. In our study, 3 patients (12%) developed strictures. Strictures were not due to tumour.

## 5. Conclusions

The ureteroscopic treatment of UTUCs is technically possible and should be undertaken in patients with imperative indication. Nevertheless a tumour free status can be achieved only rarely. The complications rate is low. Progression rate is very low for low-grade UTUCs. However, the extension of indication on non imperative cases can be a problem, because the overall recurrence rate is unclear and because the biopsy cannot always indicate the right tumour grade.

## Figures and Tables

**Table 1:** 

Number of patients	*n* = 25
Number of ureteroscopies	*n* = 288

Complications	

3/25	Permanent ureter strictures (12%)
11/288	Bleeding required blood transfusion (3.8%)
1/25	Septicaemia with *Klebsiella *
1/25	Ureteral demolition
